# PD-L2 Expression in Breast Cancer Promotes Tumor Development and Progression

**DOI:** 10.1155/2024/3145695

**Published:** 2024-07-02

**Authors:** Yuling Sun, Jie Yang, Yachun Chen, Yundi Guo, Jian Xiong, Xuqin Guo, Yawen Zhang, Li Gu, Min Tong, Weipeng Wang, Jing Sun

**Affiliations:** ^1^ Jiangsu Province Engineering Research Center of Molecular Target Therapy and Companion Diagnostics in Oncology Suzhou Vocational Health College, Suzhou 215009, China; ^2^ Center for Drug Metabolism and Pharmacokinetics College of Pharmaceutical Sciences Soochow University, Suzhou 215123, China

## Abstract

**Background:**

This work focused on investigating the role of programmed death ligand 2 (PD-L2) in the progression of breast cancer by utilizing breast cancer specimens and cells.

**Materials and Methods:**

The serum levels of soluble PD-L2 (sPD-L2) in breast cancer patients and healthy individuals were analyzed by means of the enzyme-linked immunosorbent assay, and the PD-L2 levels within 416 resected breast cancer specimens were assessed through immunohistochemistry. Concurrently, in vitro cell experiments and in vivo animal experiments were carried out to analyze the relationship between PD-L2 and the invasion and migration of breast cancer.

**Results:**

The concentration of sPD-L2 in breast cancer patients significantly increased compared to that in the control groups. Additionally, breast cancer patients with high concentrations of sPD-L2 had higher Ki67 values (≥30%) and tumor grades. PD-L2 was expressed in 79.09% of the cancer samples, which exhibited a positive correlation with the progesterone receptor (PR) and the human epidermal growth factor receptor 2 (HER2). Furthermore, we discovered that knockdown of PD-L2 inhibited the migratory and invasive abilities of both MCF-7 and MDA-MB231 cells.

**Conclusion:**

Our findings demonstrated that knockdown of PD-L2 suppressed tumor growth, providing novel insights into important biological functions.

## 1. Introduction

The programmed death ligand 2 (PD-L2), another member of the B7 family, was initially discovered as a gene differentially expressed between dendritic cells and activated macrophages [1]. PD-L2 fails to bind to the cytotoxic T lymphocyte-associated antigen (CTLA)-4 and CD28, instead, it binds to PD-1, the B7-H1/PD-L1 receptor [2]. PD-L2 expression can be induced on the surfaces of dendritic cells, macrophages, certain B cell populations, and mast cells [3, 4, 5, 6]. Moreover, it is widely suggested that PD-L2 binds to the PD-1 coinhibitory receptor to suppress immunity [4, 7]. Nonetheless, PD-L2 is also discovered to trigger T-cell growth and the generation of cytokines, even in non-PD-1 binding PD-L2 mutants and PD-1-deficient T-cells [8, 9, 10]. Therefore, there is a general consensus that PD-L2 has a synergistic promoting or inhibiting activity. In recent years, numerous reports have revealed that PD-L2 is elevated in many cancers, including ovarian cancer [11], lung adenocarcinoma [12], gastric cancer [13, 14], and esophageal squamous cell carcinoma [15]. In contrast, only a few studies have demonstrated a connection between PD-L2 and breast cancer [16, 17, 18]. Therefore, it is of significant clinical importance to explore PD-L2 expression within breast cancer.

Breast cancer, a disease with molecular heterogeneity, is the most prevalently occurring malignancy among women [19, 20, 21]. Early breast cancer, which is confined to the mammary gland or has only spread to the axillary lymph nodes, is regarded as treatable. The advancements in multimodal treatment have increased the likelihood of cure to ~70%–80% of cases [22]. However, advanced (metastatic) disease cannot be cured with the existing treatments such as surgery, radiotherapy, chemotherapy, endocrine therapy, and targeted biological therapy [23, 24]. Thus, more effective therapeutic alternatives are requisite for breast cancer. In recent years, cancer immunotherapy has provided breakthrough therapeutics for combating cancer [25, 26, 27]. Among them, anti-PD-1 or anti-PD-L1 antibodies have altered the treatment of advanced cancers, such as melanoma [28, 29], lung cancer [30], kidney cancer [31], or various others [32, 33]. However, immunotherapy has shown limited success in breast cancer, and the effect of PD-L2 on regulating breast cancer remains largely unknown [16, 17, 18].

In this study, we found that serum soluble PD-L2 (sPD-L2) concentration was elevated in breast cancer patients compared with healthy controls by enzyme-linked immunosorbent assay (ELISA), and the expression of PD-L2 was positively correlated with progesterone receptor (PR) and human epidermal growth factor receptor 2 (HER2) by immunohistochemistry experiments in breast cancer patients. We also found that PD-L2 knockdown suppressed the invasion and migration of MCF-7 and MDA-MB231 cells. Furthermore, the results of the mouse xenograft tumor assay were in line with those of the in vitro cell assays.

## 2. Materials and Methods

### 2.1. Participants

This study involved individuals from whom blood samples were obtained at the Department of General Surgery of Suzhou Municipal Hospital in Suzhou, China, during the period from January 2018 to December 2020. In addition, blood samples were also simultaneously collected from healthy blood donors to be used as controls.

Tumor tissues were acquired from breast cancer patients who underwent surgery at the Department of General Surgery of Suzhou Municipal Hospital and Kunshan People's Hospital in Jiangsu Province, China, from January 2008 to December 2018, after obtaining informed consent. There were a total of 416 tumor tissues utilized in this study, and the patients had not undergone radiotherapy and chemotherapy prior to the surgery. The stage of patients was assessed according to the 8th edition of the American Joint Committee on Cancer (AJCC) staging manual [34]. The tissues were stained with hematoxylin and eosin to confirm the pathological diagnosis. The clinical parameters were documented and can be found in Table 1. Prior to this work, the Ethics Review Board of the hospital gave its approval to our study. The tissue donors provided informed consents.

### 2.2. Cell Culture and Antibodies

We obtained two human breast cancer cell lines, namely, MCF-7 and MDA-MB231, from the Institute of Cell Biology (Chinese Academy of Sciences, Shanghai, China) and cultured them in Dulbecco's Modified Eagle Medium (DMEM) which contained 10% fetal bovine serum (FBS; HyClone, Logan, UT, USA) along with 1% penicillin/streptomycin. For the knockdown of PD-L2 (shPD-L2), we employed the lentivirus vector T7960-1-LV3R-E11 which contains an short hairpin RNA (shRNA) sequence (GenePharma, Suzhou, China). The following oligonucleotides were utilized: shNC : 5′-TTCTCCGAACGTGTCACGT-3′; shPD-L2 : 5′-GGCCAGCATTGACCTTCAAAG-3′. Cells were incubated with the medium containing lentivirus together with 5 *μ*g/mL of polybrene for 48 hr and then selected with 5.0 *μ*g/mL puromycin for 2 weeks, and the knockdown effect of PD-L2 was validated by Western blotting. All cell lines used in this study were confirmed to be mycoplasma-free and cultivated under 37°C and 5% CO_2_ conditions.

The antibodies utilized in this paper are presented as follows: Anti-PD-L2 antibody (ab200377, ab187662), Anti-HER2 antibody (ab134182), and Anti-GAPDH antibody (ab181602) were procured from Abcam, Cambridge, United Kingdom. Anti-Ki67 antibody (GM7240), Anti-ER antibody (GT2056), and Anti-PR antibody (GT2160) were ordered from Gene Tech, Shanghai, China. The secondary antibody employs a goat anti-rabbit antibody with horseradish peroxidase which was obtained from Beyotime Biotechnology, Shanghai, China.

### 2.3. Tissue Microarray Construction

For the construction of tissue microarrays, formalin-fixed and paraffin-embedded tissue blocks that contained tumor tissue were recognized by area-specialized histopathologists on hematoxylin and eosin-stained slides. Thereafter, two duplicate 0.2-mm cores were obtained from the periphery and center of the tumor and arrayed in the recipient paraffin block using the tissue puncher/arrayer (Beecher Instruments, Silver Spring, MD, USA). Subsequently, 4-*μ*m tissue sections were prepared and placed onto Superfrost Plus slides to conduct immunohistochemistry.

### 2.4. Immunohistochemistry

The following is the procedure for Ki67 staining: 4-*μ*m sections were dewaxed in xylene, then hydrated through a series of graded ethanol baths, and rinsed in water. The activity of endogenous peroxidase (GK600510, Gene Tech) was blocked. Antigen retrieval was carried out by microwaving at full power (750 W) in citrate buffer with pH 6.0 for 10 min. The MIB-1 primary antibody (GM7240, Gene Tech) was incubated for 1 hr at room temperature. All washes and dilutions were performed using phosphate-buffered saline (PBS). Biotinylated rabbit anti-mouse immunoglobulin was applied, followed by avidin–biotin complex (GK600510, Gene Tech). Diaminobenzene (DAB, GK600510, Gene Tech) was used to develop peroxidase activity, and counterstaining was done with hematoxylin. The observer (blinded to the patient outcome) examined the stained sections using a standard light microscope with a 40x objective and a 10 × 10 eyepiece graticule. The Ki67 score was defined as the percentage of the total number of tumor cells (at least 1,000) with nuclear staining in 10 high-powered fields (40x).

Estrogen receptor (ER), PR, HER2, and PD-L2 were stained using the same microwave antigen recovery staining procedure as described above. The Anti-ER antibody (GT2056, Gene Tech), Anti-PR antibody (GT2160, Gene Tech), Anti-HER2 antibody (ab134182, Abcam), and Anti-PD-L2 antibody (ab200377, Abcam) were incubated for 2 hr at room temperature. The histoscore (H-score) was used to assess ER and PR, incorporating the evaluation of the intensity of staining (0–3) and the number of cells staining (range of score 0–300). By this method, ER- or PR-positive tumors have a score of >1. For HER2 scoring, specimens were classified as positive if the immunohistochemical staining was 3+ or if the staining was 2+ and FISH (fluorescence in situ hybridization) was positive.

Data on PD-L2 expression only included tumors with adequate tissue to perform immunohistochemistry. PD-L2 expression was assessed using a scoring scheme that relied on the distribution of positive tumor cells and staining intensity. In addition, the intensity factor ranging from zero (the intensity is either negative or staining, but it just exceeds the background) to two (the slide shows strong positive or dark brown staining when examined under a microscope) was used to multiply the distribution score, which estimates the percentage of cells that were positively stained. The intensity of PD-L2 immunostaining was classified as follows: the sections that scored Grade 1 were classified as low PD-L2 positivity groups, and the sections of Grade 2 were classified as groups with high PD-L2 positivity.

The immunohistochemically stained sections were independently examined by two researchers who were not aware of the patients' clinicopathological features.

### 2.5. Western Blotting

We used cold radio-Immunoprecipitation assay (RIPA) buffer with phenylmethanesulfonyl fluoride (PMSF) to homogenize cells and then centrifuged the samples to obtain the supernatants. A total of 30 *μ*g of proteins were separated through SDS-PAGE prior to being transferred onto the 0.22-*μ*m polyvinylidene fluoride (PVDF) membrane. The membrane was blocked with a blocking buffer (5% defatted milk within tris-buffered saline tween-20 (TBST) that is composed of 10 mM Tris-HCl pH 8.0, 0.05% Tween 20, and 150 mM NaCl) at ambient temperature for 1 hr. After being subjected to overnight primary antibody incubation at 4°C, the membrane was probed with horseradish peroxidase-labeled secondary antibody at ambient temperature for 1–2 hr. The enhanced chemiluminescence (ECL) Western blot detection reagents (Millipore) were utilized to visualize the membranes.

### 2.6. Cell Survival Assay

Cells (shNC, shPD-L2) were seeded in 96-well plates at a density of 3 × 10^3^ cells/well in triplicate and maintained in an incubation state overnight. Cell viability was determined with the Cell Counting Kit-8 assay (CK04, Dojindo) in accordance with specific protocols. Each clone was tested in at least three replicate experiments. The data were measured in terms of the growth percentage in comparison with the controls. The average was calculated using three or more replicates for each clone. The results were represented in the form of the growth proportion in comparison with the controls.

### 2.7. Wound Healing Assay

Cells (shNC, shPD-L2) were plated in 6-well plates at a density of 5 × 10^5^ cells per well and allowed to grow until they reached 80% confluence. The cells were scratched in a straight line using a 200-*μ*l sterile pipette tip, then washed three times with PBS, and cultured for 24 hr. The scratches were visualized by a fluorescence microscope at a magnification of 200x (Nikon 80I; Nikon Corporation) and analyzed using Image J software (1.80v; National Institutes of Health).

### 2.8. Cell Migration and Invasion Assays

The Transwell migration assay was carried out by using Transwell inserts (24-well plates, Corning Costar) with a filter having an 8 *μ*m pore. After trypsinization into single cells, 5.0 × 10^4^ cells (shNC, shPD-L2) were inoculated on top of the Transwell chamber which contained a serum-free medium. The bottom chamber was filled with 20% FBS-containing medium. After 12–16 hr, the cells were fixed with methanol on the insert membranes and stained with Giemsa solution, and nonmigrating cells on the upper surface of the membrane were gently removed. The number of migrated cells was counted in five randomly chosen fields per insert (x200) using Image J software (1.80v; National Institutes of Health).

In the cell invasion assay, the upper chamber was first coated with Matrigel before cell seeding.

### 2.9. Tumor Xenograft Model

This work obtained nude mice from Charles River in Beijing. The animal experimental protocols obtained approval from the Institutional Animal Ethics Committee of Suzhou Vocational Health College, China. The animals were randomly divided into two groups (*n* = 6 for each). Mice were subjected to subcutaneous inoculation with MDA-MB231-NC and MDA-MB231-shPD-L2 cells (2 × 10^6^) suspended in a serum-free medium (100 *μ*L). Approximately 1 week later, the average tumor volume was 400–500 mm^3^. Thereafter, the tumor size was determined using the Vernier caliper at 2-day intervals, and the tumor volume (mm^3^) was calculated by multiplying the length by the square of the width and dividing by two. On the 15th day, the tumors were harvested.

### 2.10. Statistical Analysis

All data are expressed as the mean ± SEM. Statistical analysis was carried out using GraphPad Prism 8.0 software (GraphPad Software, La Jolla, CA, USA) through Student's *t*-test or two-way ANOVA. The status of proteins related to the main clinical and pathologic features was compared by means of *χ*^2^ tests and Fisher's exact test when necessary. *P* < 0.05 indicated statistical significance.

## 3. Results

### 3.1. sPD-L2 Is Overexpressed in the Serum of Breast Cancer Patients

**Figure 1:**
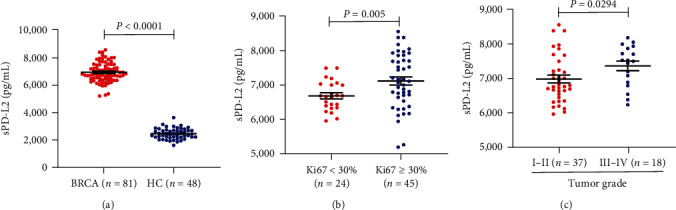
Serum PD-L2 expression is increased in breast cancer: (a) increased sPD-L2 was observed in the sera from 81 patients with breast cancer (BRCA, 6926.45 ± 78.38 pg/mL) compared to 48 healthy controls (HCs, 2452.83 ± 57.53 pg/mL) (*P* < 0.0001), (b) sPD-L2 concentrations were higher in patients with breast cancer with high Ki67 expression (*P*=0.005), (c) comparison of sPD-L2 in tumor grade in patients with breast cancer (*P*=0.0294).

To assess the relationship between the PD-L2 level and breast cancer, serum sPD-L2 levels were detected among healthy controls and breast cancer patients by means of ELISA. As depicted in Figure 1(a), the concentration of sPD-L2 was increased among breast cancer patients (6926.45 ± 78.38 pg/mL) as compared to that in healthy controls (2452.83 ± 57.53 pg/mL; *P* < 0.0001). Moreover, breast cancer patients with high concentrations of sPD-L2 had higher Ki67 values (≥30%) (Figure 1(b)) and tumor grade (Figure 1(c)). As a nuclear protein, the expression of Ki67, which is measured via immunohistochemistry, is a marker of proliferation. In conclusion, PD-L2 is likely to be associated with the occurrence of breast cancer.

### 3.2. PD-L2 Expression in Breast Cancer Tissues

**Figure 2:**
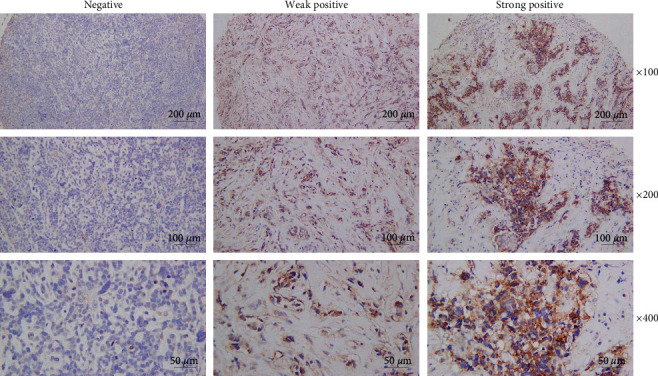
PD-L2 expression in breast cancer detected by immunohistochemistry. Breast cancer tissue microarray immunohistochemical staining of PD-L2: (left) negative, (middle) weakly positive, and (right) strongly positive.

Subsequently, in order to clarify the relationship of PD-L2 with the progression of breast cancer, the PD-L2 levels within tumors were analyzed. Immunohistochemistry disclosed that PD-L2 was positively expressed in 329 (79.09%) out of 416 tumors, was lowly expressed in 192 (46.15%), and was highly expressed in 137 (32.93%) of the tumors (Figure 2). The immunohistochemical results indicated that PD-L2 mainly resided on the cancer cell membrane and in the cytoplasm.

### 3.3. Correlations between PD-L2 Level and Clinicopathological and Molecular Characteristics

Table 1 summarizes the patient characteristics based on the PD-L2 expression status. The PD-L2 levels in breast cancer samples were examined, and as a result, it showed a positive correlation with HER2 (*P*=0.0179) and PR (*P*=0.0123). Taken together, PD-L2-positive tumors exhibited biological aggressiveness.

However, the PD-L2 expression was not related to additional clinicopathological factors like age, tumor size (*P*=0.4088), tumor grade (*P*=0.3513), lymph node metastasis (*P*=0.3508), or estrogen receptor expression (*P*=0.1769).

### 3.4. PD-L2 Silencing Impeded MCF-7 and MDA-MB231 Cell Growth

**Figure 3:**
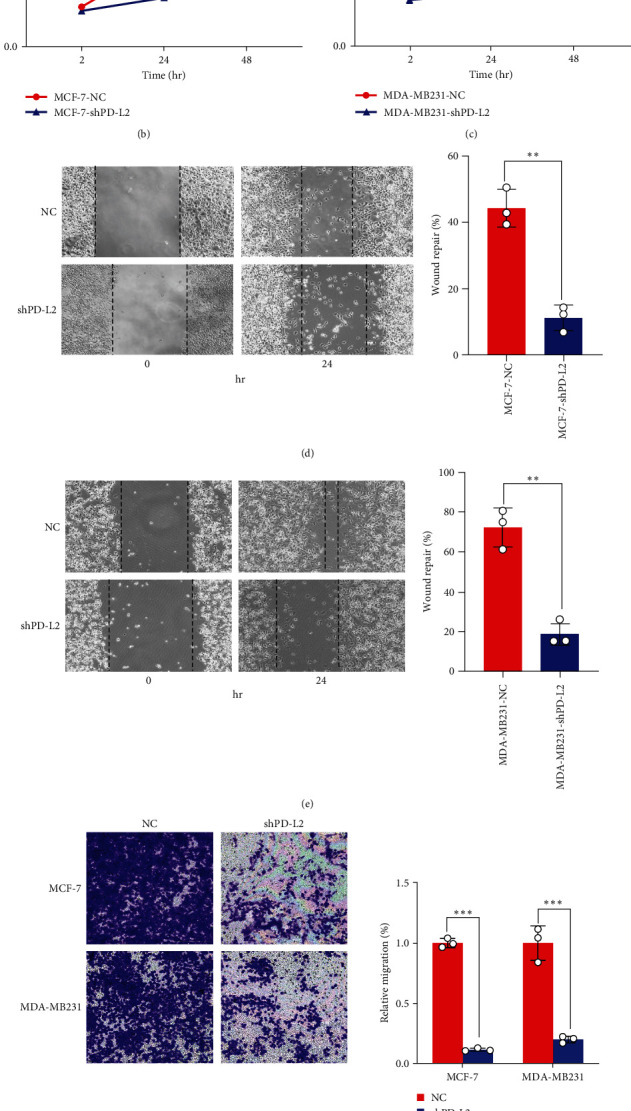
PD-L2 deficiency inhibits tumor metastasis: (a) PD-L2 was knocked down in MCF-7 and MDA-MB231 cells. Western blot analysis of the protein levels of PD-L2 ( ^*∗∗∗*^*P* < 0.001). Knockdown of PD-L2 inhibited the proliferation of MCF-7, (b) and MDA-MB231, (c) cells ( ^*∗∗∗*^*P* < 0.001). Wound healing assay in control and PD-L2 knockdown with MCF-7, (d) and MDA-MB231, (e) cells as detected by wound healing assay. Images were taken 0 and 24 hr after wound formation. The right part of the figure shows the percentage of wound repair ( ^*∗∗*^*P* < 0.01). Representative images of Transwell assay for cell migration, (f) and invasion, (g) in MCF-7 and MDA-MB231 cells. The right part of the figure shows the relative percentage compared to the negative control ( ^*∗∗∗*^*P* < 0.001).

To explore the potential biological effect of PD-L2 on breast cancer cells, PD-L2 knockdown MCF-7 and MDA-MB231 stable cells were constructed. Gene knockdown efficiency of shPD-L2 lentiviral vector was confirmed by Western blotting (Figure 3(a)). The growth curve indicated that PD-L2 knockdown impaired the proliferation of MCF-7 and MDA-MB231 cells (Figures 3(b) and 3(c)). As revealed by the scratch assay, PD-L2 knockdown MCF-7 and MDA-MB231 cells had less migration than empty vector-transfected counterparts (Figures 3(d) and 3(e)). Consistently, the Transwell assay showed that PD-L2 knockdown significantly decreased migration of both cell lines (Figure 3(f)). The invasion assay demonstrated that PD-L2 knockdown blocked the invasion of these two cell lines (Figure 3(g)). In total, low PD-L2 levels inhibit breast cancer cell proliferation, migration, and invasion.

### 3.5. PD-L2 Knockdown Inhibited Tumor Growth In Vivo

**Figure 4:**
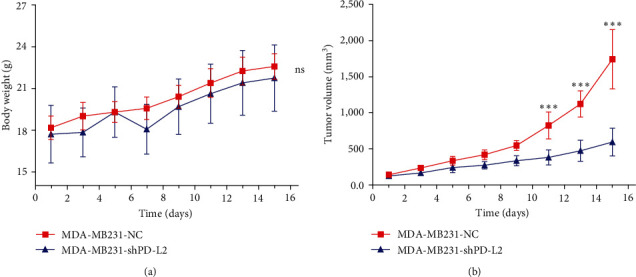
PD-L2 knockdown suppress tumor growth in the xenograft mouse model: (a) MDA-MB231 cells (either NC or shPD-L2) were subcutaneously injected into mice. The weights of the mice were measured every 2 days (*n* = 6) (ns: No significance), (b) Tumor growth was measured every 2 days (*n* = 6) ( ^*∗∗∗*^*P* < 0.001).

Subsequently, we evaluated the function of PD-L2 in tumor growth in vivo. Animals were subjected to subcutaneous injection of MDA-MB231-shPD-L2 and MDA-MB231-shNC cells. The tumor volume was observed at 2-day intervals. The results indicated that there was no significant difference in body weight between the two groups (Figure 4(a)). In addition, tumors with PD-L2 knockdown showed a significantly decreased growth rate compared to the controls (Figure 4(b)).

## 4. Discussion

PD-L2 was discovered to be a new member of the B7 family among the genes that were differentially expressed in dendritic cells and activated macrophages in a gene library [1]. Initially, it was thought that PD-L2 expression was restricted to antigen-presenting cells such as dendritic cells and macrophages [3, 4, 5, 6]. Nonetheless, it has now been discovered that PD-L2 is expressed in various tumor, stromal, and immune cells, according to microenvironmental stimuli [35]. Compared with PD-L1, PD-L2 displays the higher affinity to PD-1 [36]. As Tanegashima et al. [37] discovered, the individual or simultaneous expression of PD-L2 with PD-L1 in cancer cells inhibited antitumor immunity, which was related to the resistance to anti-PD-L1 treatment that was found in preclinical animal models. The mechanisms by which PD-L2 modulates tumor immunity are still unclear, but these results suggest the importance of PD-L2 for evading antitumor immunity. Furthermore, the blockade of PD-L1 and PD-1 or PD-L2 should be taken into consideration in order to attain the optimal immunotherapy for PD-L2-positive tumors. PD-L2 is highly expressed in various human cancer types, such as ovarian cancer [11], lung adenocarcinoma [12], gastric cancer [13, 14], and esophageal squamous cell carcinoma [15]. By conducting immunohistochemistry, we demonstrated that positive PD-L2 expression was presented in 329 (79.09%) out of 416 breast cancer samples, weak expression was seen in 192 (46.15%) of the patients, and strong expression was observed in 137 (32.93%) of the patients.

sPDL2 is mainly thought to be produced by the cleavage of membrane-bound PD-L2, similar to its cousin PD-L1 [38]. According to a recent study, the expression of sPD-L2 significantly increased among nonsmall cell lung cancer patients when compared to that of normal donors [39]. In another study, it was found that platinum resistance in advanced epithelial ovarian carcinoma was associated with sPDL2 [11]. Therefore, sPD-L2 is capable of enhancing cancer invasion via its interaction with the membrane-bound PD-1 on immune cells [35]. Based on our results, by means of ELISA, the serum sPD-L2 expression increased among breast cancer patients. The sPD-L2 level among the patients with breast cancer was 6926.45 ± 78.38 pg/mL, while the concentration in healthy controls was 2452.83 ± 57.53 pg/mL. Moreover, the patients with breast cancer having high concentrations of sPD-L2 had higher Ki67 values (≥30%) and tumor grades. These findings imply that sPD-L2 can serve as a biomarker for the diagnosis and prognosis prediction of breast cancer.

The upregulation of PD-L2 has been noticed to be associated with the poor prognostic outcome of specific tumor types, while different results are uncovered in additional cancer types. Hence, it is of great importance to examine the precise effect of PD-L2 on tumor tissue. In our study, the PD-L2 expression significantly increased in 79.09% of breast cancer tissues, demonstrating a notable correlation with HER2 and PR. Nevertheless, the PD-L2 expression was not related to other clinicopathological factors such as age, tumor size (*P*=0.4088), tumor grade (*P*=0.3513), lymph node metastasis (*P*=0.3508), or estrogen receptor expression (*P*=0.1769). This is not in line with the findings in serum where the expression levels of sPD-L2 correlate with the tumor grade. Such a discrepancy in the results is likely associated with the differences in patient basic characteristics as well as the PD-L2 immunohistochemical methods, thresholds for positive expression, and scoring systems. For instance, in studies regarding PD-L2 and tumors, positive PD-L2 expression shows significantly different prognostic significance [40, 41, 42].

Our study possesses certain limitations. Firstly, we had a relatively small number of clinical samples and relatively few indicators for analyzing PD-L2 and clinicopathological features. Secondly, the expression profiles of PD-L2 and sPD-L2 were obtained from two separate sample banks. We will continue to gather clinical samples by using tissue-matched plasma samples prior to surgery in order to assess the correlation between the tissue expression of PD-L2 and the serum expression of sPD-L2 with the clinicopathological features of the patients.

## 5. Conclusion

**Figure 5:**
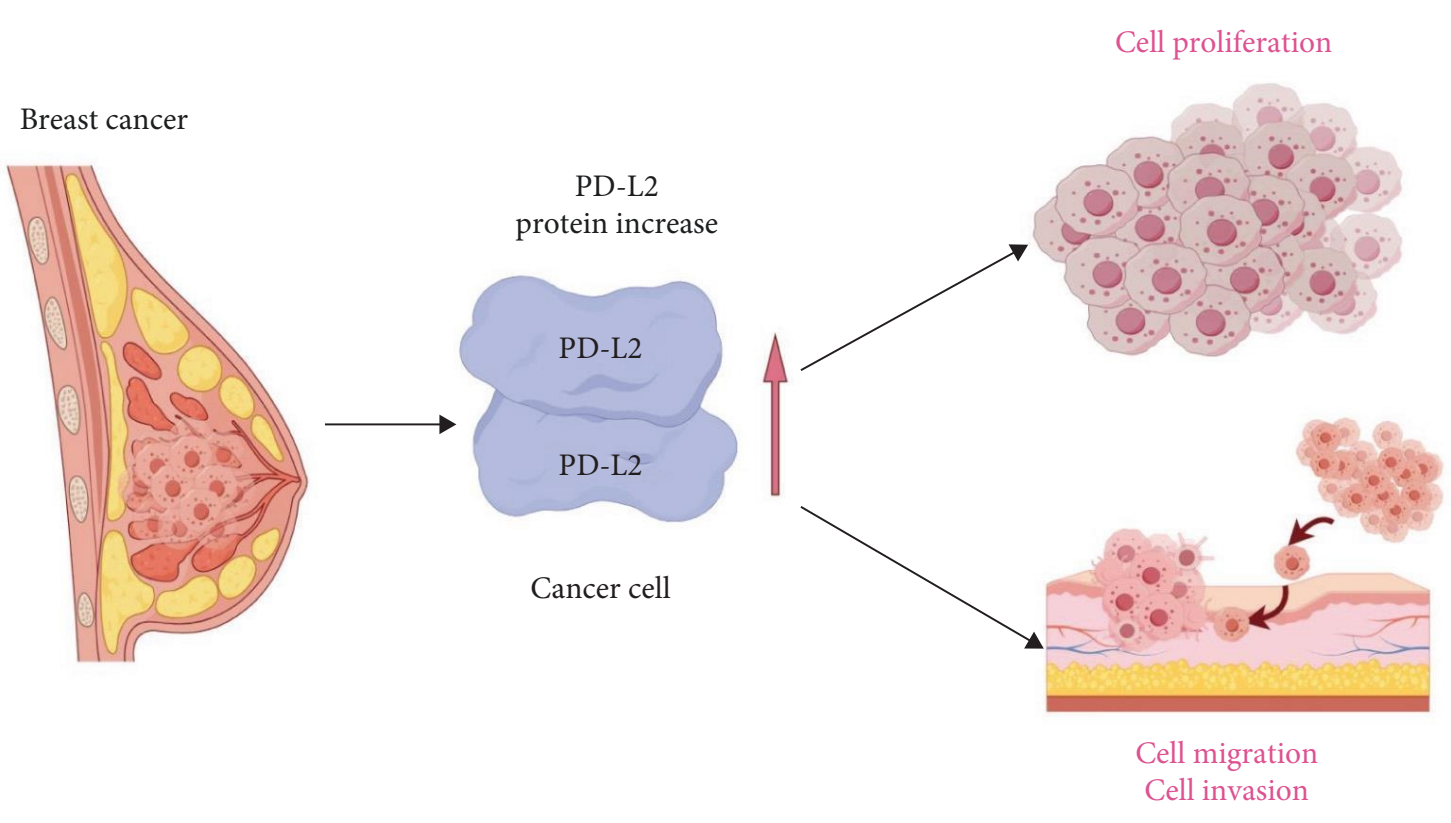
Regulatory role of PD-L2 in breast cancer. High expression of PD-L2 in breast cancer cells promotes cell growth, invasion, and migration.

To summarize, we determined the oncogenic impact of the immune checkpoint PD-L2 on the progression of breast cancer. The high expression of PD-L2 within breast cancer cells boosts cell growth, invasion, and migration. Correspondingly, knocking down PD-L2 inhibited tumor cell growth, migration, and invasion both in vivo and in vitro (Figure 5). Given the extensive upregulation of PD-L2 within breast cancer and its pro-oncogenic effect on the development of breast cancer, targeting PD-L2 represents a potential antibreast cancer treatment.

## Figures and Tables

**Table 1:** Patient features based on PD-L2 expression.

Factor	No. of patients (%)		*P*
	Low PD-L2 expression	High PD-L2 expression	
Age (y)			
Median	55	55	—
Range	26–89	27–80	—
Tumor size			
<3 cm	64 (31.53%)	42 (20.69%)	—
≥3 cm	64 (31.53%)	33 (16.26%)	0.4088
Tumor grade			
I–II	74 (36.27%)	48 (23.53%)	—
III–IV	55 (26.96%)	27 (13.24%)	0.3513
Lymph node metastasis			
Negative	18 (24.32%)	22 (29.73%)	—
Positive	19 (25.68%)	15 (20.27%)	0.3508
Tumor region			
Unilateral breast	133 (61.57%)	79 (36.57%)	—
Bilateral breast	3 (1.39%)	1 (0.46%)	0.6148
HER2			
Negative	51 (24.17%)	19 (9.00%)	—
Positive	79 (37.44%)	62 (29.38%)	**0.0179**
ER			
Negative	48 (22.43%)	22 (10.28%)	—
Positive	85 (39.72%)	59 (27.57%)	0.1769
PR			
Negative	55 (28.50%)	22 (11.40%)	—
Positive	62 (32.12%)	54 (27.98%)	**0.0123**

*Abbreviations*. PD-L2, Programmed death ligand 2; HER2, Human epidermal growth factor receptor 2; ER, Estrogen receptor; PR, Progesterone receptor. Bold values are statistically significant.

## Data Availability

Data that support our results can be obtained from corresponding authors on request.

## Additional Points

*Clinical Significance*. This study shows that sPD-L2 is highly expressed among breast cancer patients and is associated with the tumor grade, demonstrating the feasibility of sPD-L2 expression as a diagnostic indicator of breast cancer. Therefore, sPD-L2 is a potential prognostic biomarker, providing a new antibreast cancer therapeutic strategy.
